# On Quantum Relations

**DOI:** 10.3390/e28050522

**Published:** 2026-05-05

**Authors:** François Dubois, Zeno Toffano

**Affiliations:** 1 LMSSC Laboratory, Conservatoire National des Arts et Métiers, 75141 Paris, France; 2 Association Française de Science des Systèmes (AFSCET), 92260 Fontenay-aux-Roses, France; 3 Laboratoire Signaux et Systèmes, UMR 8506, CentraleSupélec, Université Paris-Saclay, CNRS, 91190 Gif-sur-Yvette, France; zeno.toffano@centralesupelec.fr

**Keywords:** quantum-like modeling, quantum relations, fractaquantum, eigenlogic, quantum semantics, social laser, relational ontology, 03.65.–w, 05.65.+b, 81P99, 93B99

## Abstract

This contribution proposes a conceptual framework for quantum relations understood as operator-based, scale-dependent semantic structures. It explores the “fractaquantum” hypothesis, emphasizing that nature exhibits quantum properties at all scales, from subatomic particles to social structures. Using Pauli operators, we propose a semantic theory of quantum relations based on the “semiotic square” and on eigenlogic. The "two one-half spin" quantum composition defines the exchange operator at the basis of fundamental quantum relations. The approach is applied to macroscopic phenomena such as “social lasers” and the rhythmic “breathing” of entanglement, suggesting that individuality and social coherence are governed by scale-invariant quantum principles. This project aims to unify several quantum-like approaches under a common relational paradigm and highlights the role of fractal scaling, contextuality, non-commutativity, exchange, indistinguishability and entanglement in the emergence of semantic relations across physical, cognitive, social and artistic domains.

## 1. Introduction

The traditional view of physics maintains a strict dichotomy: the microscopic world is governed by quantum mechanics, while the macroscopic world is classical. Nature manifests itself in two ways. On the one hand, it is fractal, identical to itself at various scales, retaining the same qualitative properties over a whole range of spatial scales. On the other hand, in the infinitely small, it is quantum, and quantum mechanics is the appropriate model for predicting the outcome of experiments.

The fractaquantum hypothesis [1,2] states that the general principles of quantum mechanics should be applied at all spatial scales. It chooses to develop a worldview based on the universality of the quantum approach and imagines that the world is fractal, analogous to itself at all spatial scales. The assumption that the world is both quantum and fractal implies that quantum properties such as indistinguishability, superposition, and entanglement are scale-invariant. The fractaquantum hypothesis posits that quantum structures are not confined to microscopic physics but appear at multiple scales, including biological, cognitive, and social systems. Relations, rather than material constituents, constitute the primary ontological elements. The fractaquantum hypothesis becomes a possible approach for the description of a first approximation of nature, an asymptotic development of increasingly precise models. Of course, it does not describe a possible “breaking of scale invariance”, which could then enable us to build a more precise, “second-order” theory.

Traditionally, the Ehrenfest theorem [3] is used to describe how the expectation values of quantum observables evolve according to classical laws of motion, providing a limit where quantum systems begin to look classical. Here, we suggest that the classical appearances we observe are conditioned to our specific methods of questioning nature and on the observer–observed relationship.

Quantum theory has progressively expanded beyond its original physical domain into logic, cognition, linguistics, and social sciences. This expansion raises a foundational question: are these applications metaphorical or do they reveal a deeper relational structure common to diverse domains? In this paper, we consider a quantum-like approach [4] where quantum theory provides a general framework for understanding relations rather than objects and where this framework exhibits fractal scale-invariant features.

From a relational perspective, classical identities appear when relations stabilize at a given scale and become effectively separable, but this separability remains context-dependent and fragile. We can extend the relation concept by means of a mediator of relations. The mediator becomes a fractaquantum generalization of the physical notion of the intermediate boson. In this way, relations are generated by bosons and are structured according to a scheme that adapts with the scale.

A key challenge in modeling quantum relations is the bridge between formal syntax (the mathematical state) and semantics (the meaning of the state). The eigenlogic quantum-like approach [5] provides an operator-based model. Using Pauli matrices, this framework allows for a multi-modality logic where logical operations are represented as operators in Hilbert space, truth values correspond to eigenvalues, while meanings correspond to eigenspaces. The syntactic structure of logic is encoded in the algebra of the operators, while semantics emerges through measurement-like processes. This duality provides a natural bridge between formal logic and quantum operators. This duality suggests that quantum-like relations are simultaneously physical interactions and logical events.

A central concept underlying both the fractaquantum hypothesis and quantum-like models of social coherence is that of quantum indistinguishability. In standard quantum physics, indistinguishability refers to the impossibility of assigning persistent identities to identical particles, leading to bosonic or fermionic statistics. In the fractaquantum framework, this notion is generalized beyond microscopic particles to relational structures across different scales.

In a quantum-like approach, if we allow ourselves to imagine that human beings can be analyzed by quantum theory then we need to investigate indistinguishability as some sort of identity that exists between all human beings to the point of imagining them interchangeable. Quantum indistinguishability clearly contradicts our everyday experience of the difference existing between two individuated beings. But it does not call into question the hypothesis of a quantum nature at all scales.

This perspective finds a natural extension in quantum-like social models. In Andrei Khrennikov’s social laser framework [6,7], which applies the physics of stimulated emission to human social behavior, agents become effectively indistinguishable once they occupy the same informational or semantic state. Collective coherence emerges not from individual traits but from the indistinguishable participation of agents in a shared cognitive mode, analogous to bosonic occupation of a quantum state. In a “fractaquantum” view, the indistinguishability of individuals in a crowd, where exchange behavior is strong, could allow for the emergence of a coherent “new being” characterized by emotional contagion and libidinal ties [2] analogous to social laser actions.

Entanglement describes non-separable relations that cannot be reduced to individual components. Entanglement can be considered as the ultimate “quantum relation”: it represents a state where parts cannot be described independently. This concept extends naturally to cognitive and social individuality. Svozil describes the “breathing” in and out of entanglement as a fundamental process of individuality [8]. In the fractaquantum model, entanglement is seen in cell division, embryology, and human relationships.

In the following paragraphs, we propose a few examples on a macroscopic scale, where the mediator becomes a familiar part of our daily lives.

This paper is organized through the following sections that describe different aspects of this global approach. The ambition here is not to deliver a complete self-consistent theory but to outline how the different topics presented hereafter contribute to our hypothesis. After this introduction and a thorough discussion starting from the fractaquantum hypothesis, in Section 2 we discuss relational structures in general through some archetypal structures that we name: mediator, conversation, dance, love, pregnancy, loops, and graphs. Section 3 presents the quantum-like semantic relation approach, which is founded on the quantum operator eigenlogic method using one-half spin Pauli matrices. In Section 4, we explicitly discuss the quantum composition of two one-half spins leading to the fermion exchange operator. Section 5 discusses the central effects of exchange and indistinguishability that can be used to analyze collective effects such as the crowd and the recently proposed social laser model. Section 6 discusses some applications in the social sciences and performing arts. Section 7 concludes this discussion and opens to future developments.

## 2. Structures

We know that the concept of an object is challenged by quantum physics. The simplest atom, the hydrogen atom, is the result of the combined effect of electromagnetic interaction between a proton and an electron and of quantification. Without this interaction, the hydrogen atom would not exist. Following Heisenberg [9], we “could not foresee that atoms are neither things nor objects” and we will not attempt here to revisit Bitbol’s philosophical essay [10] which considers interdependent elements in terms of the link that connects them. Nevertheless, we consider relationships connecting elements of matter as fundamental. We begin with the interaction of two elements, the “mediator”; then we consider trios where three elements and their three pairwise relationships give rise to loops. We freely use graph theory to attempt the most general description possible.

### 2.1. Mediator

If we consider two identical quantum elements, they are indistinguishable, as Pauli’s exclusion principle, generalized with the spin-statistic theorem or symmetrization postulate, states. See, for example, the treatise by Cohen-Tannoudji, Diu and Laloë [11] stating that “only the anti-symmetric combination is possible”. The essential point for us is that the association of two identical quanta of one-half spin creates a boson of spin zero of “pure relation” if the two starting objects are identical. The association of two electrons will give rise to a “relation particle”, naturally carrying an interaction at a more elaborate level of structure. Within the fractaquantum approach, we aim to extend the notion of a relationship between two entities: a fleeting or a lasting relationship, made up of “one half plus one half” that creates the mediator of an interaction. For us, a fractaquantum mediator, or more simply a mediator, is the composition of two closely related Atoms in nature. An Atom (with a capital “A”) in the fractaquantum approach is an elementary entity, an “indivisible” element of the world, a natural constituent whose qualitative properties are modified in at least one of its parts if it is divided into two [2]. It is a system with an *a priori* finite lifetime, designed to transmit information or a relationship. The fractaquantum mediator becomes the generalization of the quantum intermediate boson. In the following paragraphs, we present some examples acting on a macroscopic scale, where the mediator corresponds to familiar parts of our daily lives.

### 2.2. Conversation

**Figure 1:**
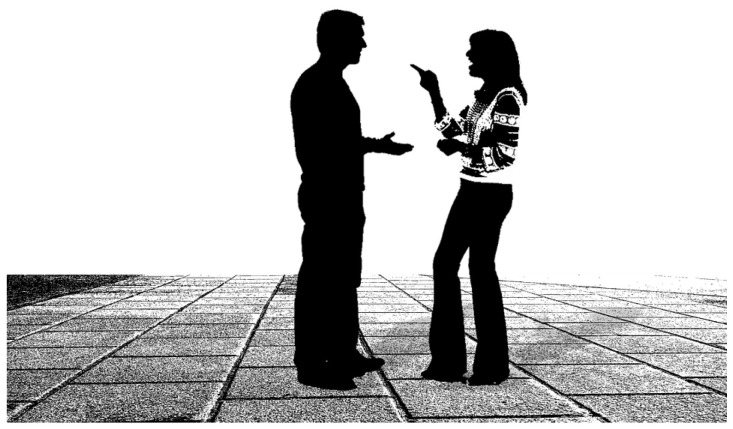
A conversation between two persons is a fractaquantum mediator [Kristin Baldeschwiler, pixabay.com, accessed on 1 August 2018].

A first example of a mediator is a simple discussion, a verbal relationship between two human beings. The internal content of the relationship is made up of the meaning of the words. It represents a physical transfer of a very small amount of matter and energy, through air vibrations during the flow of an acoustic wave. This relationship requires a certain amount of time and the investment of each person in order to ensure that the message is emitted, transmitted, and finally received. The mediator is made up of both the partners during the exchange and by the exchanged message (see Figure 1). The mediator is not limited to the content of the message, it appears at the beginning of the message and disappears once the message has been received. Its total mass, as with the intermediate boson, can be very large since the masses of both participants have to be taken into account. Its lifetime, typically a few minutes, is very small compared to the lifetimes of the two actors involved.

### 2.3. Dance

The next example goes a step further in a more intimate relationship between body and eyes, where dance evokes exchange, desire, and celebration. Dance can be seen as an elaborate form of conversation, even if it is wordless. Supported by music and a social framework, it allows for intimacy and body contact. In doing so, it creates an “intermediary being”, a structure that is stable and finite in time. People gather to admire dancing couples in Waltz, Tango, Sevillana, or Rock and Roll shows. Here we are only sketching this major theme of social encounter and creation of desire and refer the reader to the work of Schott-Billmann [12], for example.

### 2.4. Love

If you take the former relationship a step further and enter a romantic relationship, the feeling of love will animate two persons with the same desire to meet. This time, the mediator consists in the two persons united by their love. The sudden appearance of a bond is the sign of the appearance of love, as in “love at first sight”. This is reminiscent of certain coherent behaviors in electromagnetic interactions.

We will not dwell on the ephemeral nature of love because the rapid disappearance of the state of “being in love” is not inevitable, since there are also couples who stay together through feelings that last and bind the human beings. Another way of living Jacquard’s “I am the links I weave” [13]: “if he breaks the relationships he forges, every man loses the truly human part of his existence”.

As we will see in Section 4, the quantum mediator is indeed modeled by the sum of two half-spin components. In this case, the two components of the mediator are entangled. Entanglement is therefore entirely natural within the framework of the fractaquantum hypothesis. A conversation is more than just the coming together of two people: it creates meaning. Dance is not limited to the two dancers: it evokes desire and the beauty of movement. Love is not a mere collection of feelings. It is a dynamic exchange involving the body and the flesh, and it makes it possible to create life.

### 2.5. Pregnancy

Another fractaquantum mediator is created by the ability of two Atoms to merge, to integrate with each other for a certain period of time, to act as temporary carriers of a message. A very nice example of an intermediate boson, a mediator, to conclude this first attempt at formalization, is simply that of a pregnant woman. In fact, she is the union of two living beings, the woman and the foetus, which transforms from a lower-scale structure (the cell) into a small, autonomous human being. The message conveyed by this intermediary being is first of a biological nature, with the duplication of cells, then with the achievement of embryogenesis. It takes a long time (nine months) and allows life to grow from a single cell to a higher living being.

More than 20 years ago, we noted, with interest, Bernard-Weil’s reaction [14] regarding our comments on the link between the mediator and the theory of “ago-antagonistic” couples he had proposed with his colleagues [15]. The aim was to explore the notion of coupling and the search for multiple causes. In an initial approach, an ago-antagonistic couple is modeled by a system of two components, following the ideas initially proposed for prey-and-predator systems by Lotka [16] and Volterra [17]. Recall that such a mediator is itself a relationship, an interaction, a boson.

**Figure 2:**
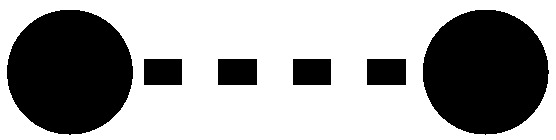
Two related Atoms form a mediator.

We can represent a mediator as an abstract graph (see Figure 2) where nature’s unbreakable elements, the Atoms, are represented by a dot and the relationship by a dotted line. It is similar in form to a Feynman diagram, except that in this first approach it is a stationary vision, stable over time, which of course limits the scope of our approach, but at least allows us to build a first model.

### 2.6. Loops

**Figure 3:**
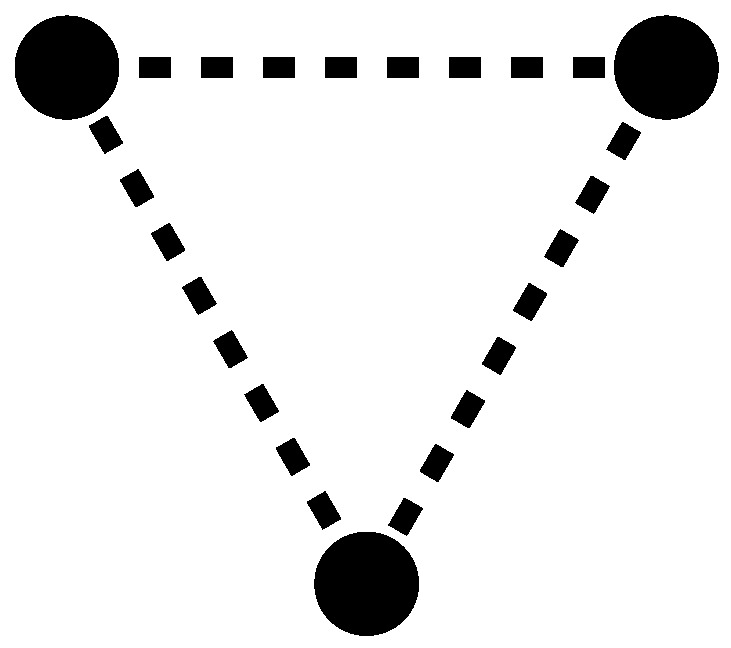
Triangular loop.

The following image shows three interacting partners with a comparable “force”. This is the case of perfect equilibrium, where the number of bosons, of relations, is equal to the number of Atoms, of fermions, as illustrated in Figure 3. In fact, if several fermions can have a pairwise relationship, the number λ of relationships of n objects linked in pairs in all possible ways is equal to the number n(n−1)/2 of combinations between these objects. Then the number of relationships is exactly equal to the number of Atoms for only three relationships, if we exclude the vacuum. We can then arrange these three elements of matter in a graph.

**Figure 4:**
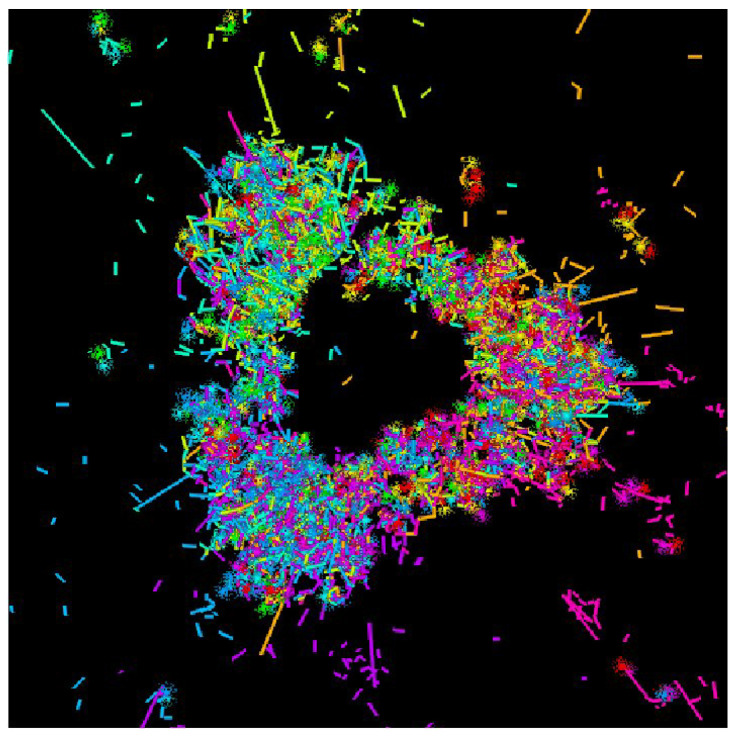
Structure of the nucleon represented in 1992 by Jean-François Colonna [18]. The three quarks at the vertices of the triangle exchange gluons, the color force, with an intensity such that the overall structure creates a loop, a true singularity of space.

An unusual example of a loop is suggested by the magnificent image of a nucleon created by Colonna [18]. This loop is the result of the continuous relationship, the permanent exchange of gluons, between the components of matter, the quarks, as illustrated in Figure 4. This forms a fairly good image of the nucleon, proton or neutron, composed of three strongly interacting quarks via a continuous exchange of gluons. Besides the impossibility of “seeing” the quarks in isolation, such a representation of the nucleon allows us to consider it at the same time as elementary, as an Atom, an unbreakable element at a certain spatial and energetic level of representation and as a composite whole structured by the means of a simple graph. Matter, within its most massive stable constituents—the nucleons—appears to be in the form of loops.

**Figure 5:**
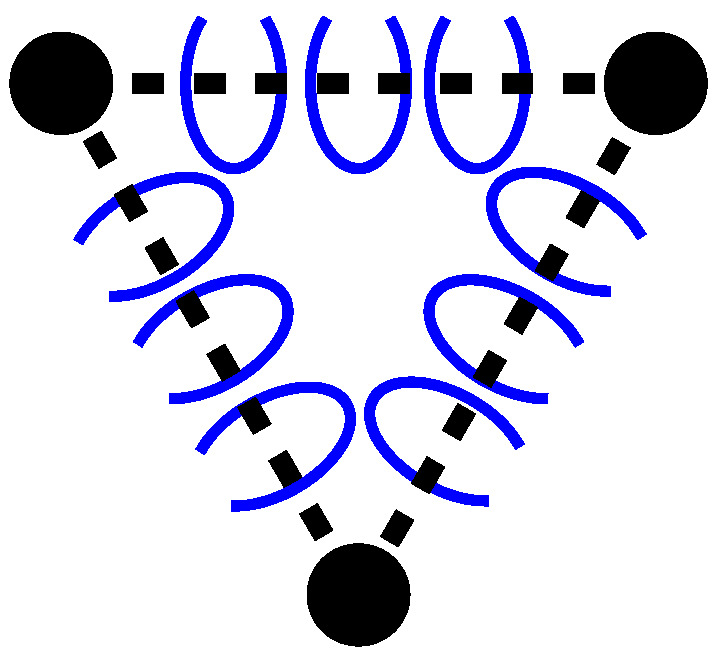
Illustration of a space that loops around connections.

Is space reduced to loops around matter? This topological structure, this quasi-continuous loop, which reflects the dynamic equilibrium of the nucleon, the fundamental constituent of matter, appears to us as a very instructive model for representing space and matter at the proton scale. We also observe that if the structure of the nucleon can be represented by a loop then there is a dual second loop formed by the outer space “around” the interacting matter. This notion is illustrated in Figure 5, from which we deduce a possible representation of space at the scale of the nucleon, made up of loops that wrap themselves around the interactions: a possible way of constructing space from loops around bosons that bind matter together, an idea inspired by loop gravity theories (see, for example, Rovelli [19]).

We should also note the generic nature of a loop representation. Starting with two identical Atoms (let us call them “H” for the sake of clarity) and another different from the first two (this time called “O”), we have two types of relationships: O–H and H–H. All in all, we have a three-link graph with one loop, the water molecule, a new Atom one step higher in the scale of spatial dimension.

### 2.7. Graphs

**Figure 6:**
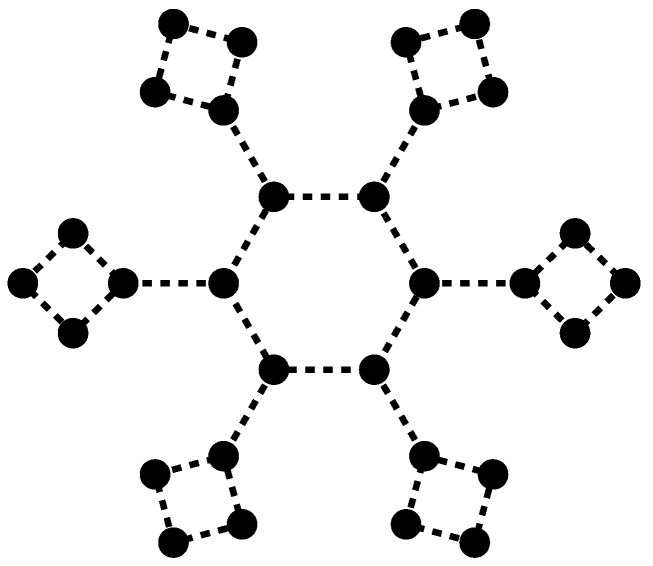
Graph structure on a given scale, consisting of 30 vertices (Atoms), 36 edges (bosons); seven cycles emerge.

For an assembly of more than three Atoms, what kind of structure emerges? We can imagine a more complex structure at a given scale, with the model of a graph of relations, of permanent exchange bosons, linking together certain Atoms of the structure. Figure 6 gives an example with n=30 Atoms and λ=36 relations. There are seven independent cycles in such a graph, loops enabling you to go from one vertex to another without retracing your steps. We are here within a familiar mathematical framework, and we refer the reader to Berge’s treatise [20].

Above all, it seems possible to formalize the existence of a structure by means of a graph made up, on the one hand, of “vertices” (Atoms) connected by links (interacting bosons). Within any given graph, we prefer structures associated with loops. So, a classical algorithm for finding a “cycle base” highlights global dynamics that can be maintained over time.

Finally, we propose to view quantum indistinguishability not through the appearances of otherness and identity, but through the identity of the relational graph underlying the structure. Let us return, for example, to Figure 6, which is already complex since it comprises 30 Atoms and 36 links. If we modify the material component of this graph, replacing the vertices by Atoms of a different nature, without changing the structure of the links between these components, we are creating a new structure, in a sense “identical” to the previous one, at least in terms of its relational graph. However, it can be distinguished from the previous one, since the Atoms that make it up are distinct. In a sense, quantum indistinguishability for complex organisms must be sought in the identity of the graph that underlies the relations, composes the structure, constitutes the being, and not in the Atoms that make up the nodes of the graph.

After this approach to structures in the physical world, we will study more abstract structures in the following section.

## 3. Quantum Semantic Relations

**Figure 7:**
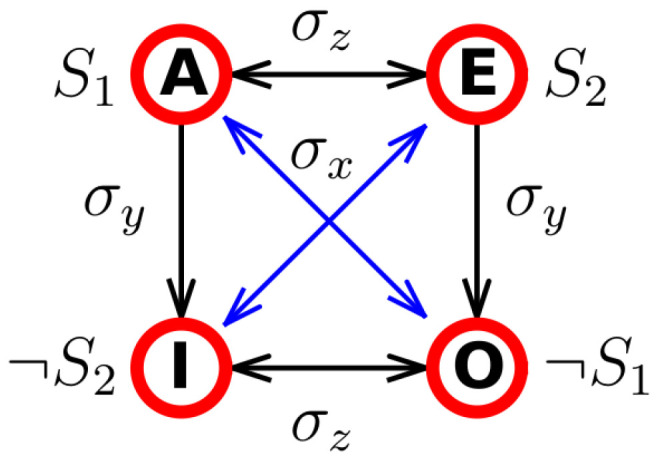
Representation of the semiotic square of opposition.

In this section, we present logical relations represented by Aristotle’s square of opposition and then its extension, by the semiotic square, in semiotics to represent semantic relations. Then a quantum-like model of the semiotic square is presented using the Pauli quantum operators. The semiotic square and the square of opposition are both conceptual diagrams used in different fields, semiotics and logic, respectively, to illustrate relationships between concepts. The square of opposition (Aristotle → Boethius → modern logic) shows logical relationships between four categorical propositions A, E, O, I based on universal and particular statements (see Figure 7):

A (Universal Affirmative): “All Q are P”;
E (Universal Negative): “No Q are P”;
I (Particular Affirmative): “Some Q are P”;
O (Particular Negative): “Some Q are not P”.

These propositions are linked by the following relations:

Contradiction: A⟷O, E⟷I. Cannot be true together and cannot be false together; are represented by two relations on the two diagonals of the square of Figure 7.
Contrariety: A⟷E. Cannot be true together but can be false together; is represented by the upper horizontal of the square of Figure 7.
Subcontrariety: I⟷O. Cannot be false together but can be true together; is represented by the lower horizontal of the square of Figure 7.
Subalternation: A⟶I, E⟶O. If A(E) is true then I(O) must also be true; are represented by two relations on the two verticals of the square of Figure 7.

Greimas and Rastier [21] extended the Aristotelian square into the semiotic square, where oppositions are not just logical but semantic relations.

This creates a dynamic field of meaning between poles $\;(S_{1}↔\neg S_{1})$. Greimas’ square thus encodes semantic motion, the semiotic “body” moving through conceptual space. The semiotic square is commonly used to model linguistic relationships such as:

- Antonymy: two words that have opposite meanings, $\;(S_{1}↔S_{2})$;
- Hyponymy: one word that is a consequence of the other, $\;(S_{1}\to \neg S_{2})$ and $\;(S_{2}\to \neg S_{1})$;
- Subcontrariety: antonymic relation between the consequents, $\;(\neg S_{1}↔\neg S_{2})$;
- Contradiction: mutually exclusive meanings, $\;(S_{1}↔\neg S_{1})$ and $\;(S_{2}↔\neg S_{2})$.

On the square of Figure 7, we find that $S_{1}$ and $S_{2}$ are antonymic (e.g., female vs. male); $S_{1}$ and $\neg S_{1}$ are contradictions (e.g., female vs. non-female). The hyponimic relations correspond to a subset relationship, e.g., to $\;(S_{1}\to \neg S_{2})$ (e.g., female implies non-man).

Galofaro et al. [22] demonstrated that the semiotic square can be represented algebraically by the Pauli matrices $\sigma_{x}$, $\sigma_{y}$, and $\sigma_{z}$ classically defined according to:

(1) $$\sigma_{x}\;\;=\;\;\left(\begin{array}{cc} 0 & 1\\ 1 & 0 \end{array}\right)\;\;,\;\sigma_{y}\;\;=\;\;\left(\begin{array}{cc} 0 & -i\\ i & 0 \end{array}\right)\;\;,\;\sigma_{z}\;\;=\;\;\left(\begin{array}{cc} 1 & 0\\ 0 & -1 \end{array}\right)\;.\;$$

This is indeed a way to represent both the semiotic square and the square of opposition using linear algebra, specifically through vector spaces and matrices providing a quantum-like extension of the square of opposition, embedding it in a Hilbert space representation.

Oppositional relations (contrary, subcontrary, contradiction, and subalternation) correspond here to the application of the Pauli matrices.

These correspond to transformations among semiotic poles: $\sigma_{z}\to$ contraries ($S_{1}$ vs. $S_{2}$); $\sigma_{x}\to$ contradictions ($S_{1}$ vs. ¬$S_{1}$); $\sigma_{y}\to$ compound transformations representing subalternation ($S_{1}⟷$ ¬$S_{2}$). The semiotic field of meaning thus maps naturally onto a two-level quantum system (a qubit).

It is interesting to note that one can travel from one node to the other in the square (see Figure 7) by successively applying the Pauli operators. This is due to the algebraic properties of the product of Pauli operators (commutation and anti-commutation) such as $\sigma_{x}\cdot \sigma_{y}=i\sigma_{z}$.

Eigenlogic [5] generalizes classical logic using linear algebra and quantum mechanics; it represents logical propositions (AND, OR, NOT) as specific operators. Logical transformations correspond to matrix operations on vectors.

Eigenlogic can be considered as a formal bridge between the fractaquantum hypothesis and the logical structuring of macroscopic systems; it also permits the extension to multi-valued logic fitting into the spectrum and measurement aspects.

The Pauli-matrix approach fits naturally within eigenlogic, creating a quantum-like model for semantics and logic. This structure creates a syntactic–semantic duality where the operator represents the syntax of the logic, while the measurement provides the semantics by the eigenvalues (truth values). Just as the Pauli exclusion principle prevents two fermions from occupying the same state to create physical structure, these operators define the boundaries and “exclusion” zones in semantic space, creating the structure of meaning. The integration of the semiotic square and Pauli operators also supports the fractaquantum hypothesis by suggesting a scale invariance because logic is not restricted to the microscopic; it operates within human language, cognition, and social structures. The relation mediator is represented here by the Pauli operators that create both matter and social structures. The act of assigning meaning to a text or a social situation is seen as a quantum measurement. This process “projects” a subject’s state onto a specific eigenvector, effectively “freezing” the potential meaning into a singular observation, as in the reduction of the wave packet.

By treating semiotic relations as quantum operators, we bridge the gap between the infinitely small and the macroscopic world of meaning. The Pauli operators provide a rigorous algebraic language to describe how logical “Atoms” (whether particles, words, or individuals) relate to one another to form coherent, multi-scale structures.

## 4. One-Half Spin Composition

We imagine two particles *a* and *b* with one-half spin. We want to build a model for the {a,b} association. In particular, we ask what is the value of the spin of the “composite” system {a,b}. The result is simply expressed, even if it leads to a curious algebra: the spin of the “integrated set” {a,b} is 0 or 1, as we will show hereafter. In short, when we add two spins, we find the relation: 1/2+1/2=0 or 1.

The essential point here is that the association of two identical quanta of one-half spin creates a “pure relation” boson of integer spin. The association gives rise to a “relation particle”, naturally carrying an interaction at a higher-level structure. Recall that in the fractaquantum approach, we aim to generalize the notion of relationship between two entities, an ephemeral or lasting relationship, made up of the “one half plus one half” that creates the mediator of an interaction.

In this section, we discuss the composition of two one-half spins using Pauli operators. This is a fundamental topic in quantum mechanics, dealt with in numerous works, such as the book by Cohen-Tannoudji et al. [11]. Our approach is based on the explicit use of the tensor product. For fundamental mathematical concepts, readers are referred to Lelong-Ferrand and Arnaudies [23] or Dixmier [24].

### 4.1. Half-Integer Spin Space

A half-spin space, denoted $\;\Sigma_{1/2}$, is a vector space over the field of complex numbers, of dimension 2, so that two linear applications $\;S_{z}\;$ and $\;S_{2}\;$ operate on the space $\;\Sigma_{1/2}$. The $\;S_{z}\;$ operator is the “spin in the O*z* direction”, and it admits a basis of eigenvectors $\;e_{\pm 1/2}$. The associated eigenvalues allow us to take both values −1/2 and +1/2:

$$S_{z}\;•\;e_{+}\;=\;\frac{1}{2}\;e_{+}\;,\;S_{z}\;•\;e_{-}\;=\;-\frac{1}{2}\;e_{-}.\;$$

Furthermore, the $\;S_{2}\;$ operator is the “square of the angular momentum”. It is proportional to the identity I on the space $\;\Sigma_{1/2}\;$:

$$S_{2}\;•\;e_{\pm }\;=\;\frac{3}{4}\;e_{\pm }.\;$$

The spin space $\;\Sigma_{1/2}\;$ corresponds to $\;j=\frac{1}{2}$; it is of dimension two. In the basis formed by $\;e_{+}\;$ and $\;e_{-}$, the $\;S_{z}\;$ and $\;S_{2}\;$ operators are represented by the following matrices:

$$S_{z}\;\;=\;\;\frac{1}{2}\;\left(\begin{array}{cc} 1 & 0\\ 0 & -1 \end{array}\right)\;\;,\;S_{2}\;\;=\;\;\frac{3}{4}\;\;I\;.\;$$

We build a “relation” between particles; the product basis states are eigenfunctions of the spin operator $\;S_{z}\;$. Then, the states satisfying the Pauli principle are simultaneous eigenfunctions of $\;S_{2}\;$; that is, eigenfunctions of an operator of all permutations. For a more detailed quantum-like approach using group operations and graph theory, one can refer to Scholes [25].

In the case of one-half spin, as well as in all other cases, even if we do not treat them explicitly here, we introduce the operators $\;S_{x}\;$ and $\;S_{y}\;$ in the other two directions of space. They are for $\;j=\frac{1}{2}$ written as

$$S_{x}\;\;=\;\;\frac{1}{2}\;\left(\begin{array}{cc} 0 & 1\\ 1 & 0 \end{array}\right)\;\;,\;S_{y}\;\;=\;\;\frac{1}{2}\;\left(\begin{array}{cc} 0 & -i\\ i & 0 \end{array}\right)\;.\;$$

It is conventional to introduce the Pauli matrices $\;\;\sigma_{x},\;\sigma_{y},\;\sigma_{z}\;\;$ defined previously in (1). For a one-half spin, we have the relations

$$S_{x}=\frac{1}{2}\;\sigma_{x}\;\;,\;S_{y}=\frac{1}{2}\;\sigma_{y}\;\;,\;S_{z}=\frac{1}{2}\;\sigma_{z}\;\;.\;$$

We can verify the following relations, which simplify the algebraic calculations that follow:

$$\left\{\begin{array}{c} {\left(\sigma_{x}\right)}^{2}\;=\;{\left(\sigma_{y}\right)}^{2}\;=\;{\left(\sigma_{z}\right)}^{2}\;=\;I\;\\ \sigma_{x}\;\sigma_{y}+\sigma_{y}\;\sigma_{x}\;=\;\sigma_{y}\;\sigma_{z}+\sigma_{z}\;\sigma_{y}\;=\;\sigma_{z}\;\sigma_{x}+\sigma_{x}\;\sigma_{z}\;=\;0\;\\ \sigma_{x}\;\sigma_{y}\;=\;i\;\sigma_{z}\;\;,\;\sigma_{y}\;\sigma_{z}\;=\;i\;\sigma_{x}\;\;,\;\sigma_{z}\;\sigma_{x}\;=\;i\;\sigma_{y}\;.\; \end{array}\right.\;$$

It is then easy to establish the relation

$$S_{2}\;=\;{\left(S_{x}\right)}^{2}\;+\;{\left(S_{y}\right)}^{2}\;+\;{\left(S_{z}\right)}^{2}\;.\;$$

### 4.2. Composition of Two One-Half Spins Using the Tensor Product

To add two one-half spins, we work in the tensor product space $\;\;\;\Sigma_{1/2}\;⊗\;\Sigma_{1/2},\;\;\;$ generated by the tensor products of the basis vectors $\;\;e_{k}⊗\varepsilon_{m}\;\;$ with k and m half-integer numbers belonging to the set $\;\mu_{1/2}=\left\{+\frac{1}{2}\;,\;-\frac{1}{2}\right\}$. In this new space, the operators $\;S_{x}^{★},\;$$\;S_{y}^{★},\;$$\;S_{z}^{★}\;$ and $\;S_{2}^{★}\;$ are calculated with a rule of “independent addition of the two angular moments”, i.e.,

$$\left\{\begin{array}{c} S_{x}^{★}\;=\;S_{x}\;⊗\;I\;\;+\;\;I\;⊗\;S_{x}\;\;,\;S_{y}^{★}\;=\;S_{y}\;⊗\;I\;\;+\;\;I\;⊗\;S_{y}\;.\\ S_{z}^{★}\;=\;S_{z}\;⊗\;I\;\;+\;\;I\;⊗\;S_{z}\;\;,\;S_{2}^{★}\;=\;{\left(S_{x}^{★}\right)}^{2}\;+\;{\left(S_{y}^{★}\right)}^{2}\;+\;{\left(S_{z}^{★}\right)}^{2}\;. \end{array}\right.\;$$

We observe that $\;S_{x}^{★}$, $\;S_{y}^{★}\;$ and $\;S_{z}^{★}\;$ are “non-local” operators, from the point of view of entanglement (see, for example, the article by Aspect [26]). From a mathematical point of view, they are not in the form of a tensor product like A⊗B.

Then, we have the matrix relations

$$\left(\sigma_{x}\;⊗\;I\right)\;=\;\left(\begin{array}{cc} 0 & I\\ I & 0 \end{array}\right)\;,\;\left(I\;⊗\;\sigma_{x}\right)\;=\;\left(\begin{array}{cc} \sigma_{x} & 0\\ 0 & \sigma_{x} \end{array}\right)\;,\;\left(\sigma_{y}\;⊗\;I\right)\;=\;\left(\begin{array}{cc} 0 & -i\;I\\ i\;I & 0 \end{array}\right)\;$$

$$\left(I\;⊗\;\sigma_{y}\right)\;=\;\left(\begin{array}{cc} \sigma_{y} & 0\\ 0 & \sigma_{y} \end{array}\right)\;,\;\left(\sigma_{z}\;⊗\;I\right)\;=\;\left(\begin{array}{cc} I & 0\\ 0 & -I \end{array}\right)\;,\;\left(I\;⊗\;\sigma_{z}\right)\;=\;\left(\begin{array}{cc} \sigma_{z} & 0\\ 0 & \sigma_{z} \end{array}\right)\;.\;$$

For more information about this calculus, we refer the reader to the book of Pauncz [27]. Then, we have

$$S_{x}^{★}\;=\;S_{x}\;⊗\;I\;\;+\;\;I\;⊗\;S_{x}\;=\;\frac{1}{2}\;\left(\begin{array}{cc} \sigma_{x} & I\\ I & \sigma_{x} \end{array}\right)\;,\;\;S_{y}^{★}\;=\;S_{y}\;⊗\;I\;\;+\;\;I\;⊗\;S_{y}\;=\;\frac{1}{2}\;\left(\begin{array}{cc} \sigma_{y} & -i\;I\\ i\;I & \sigma_{y} \end{array}\right),\;$$

$$S_{z}^{★}\;=\;S_{z}\;⊗\;I\;\;+\;\;I\;⊗\;S_{z}\;=\;\left(\begin{array}{cccc} 1 & 0 & 0 & 0\\ 0 & 0 & 0 & 0\\ 0 & 0 & 0 & 0\\ 0 & 0 & 0 & -1 \end{array}\right)\;.\;$$

### 4.3. One-Half Spin Square, Swap and Exchange Operators

We evaluate the squares of these matrices, and we get

$${\left(S_{x}^{★}\right)}^{2}\;=\;\frac{1}{2}\;\left(\begin{array}{cc} I & \sigma_{x}\\ \sigma_{x} & I \end{array}\right)\;,\;\;{\left(S_{y}^{★}\right)}^{2}\;=\;\frac{1}{2}\;\left(\begin{array}{cc} I & -i\;\sigma_{y}\\ i\;\sigma_{y} & I \end{array}\right)\;,\;\;{\left(S_{z}^{★}\right)}^{2}\;=\;\left(\begin{array}{cccc} 1 & 0 & 0 & 0\\ 0 & 0 & 0 & 0\\ 0 & 0 & 0 & 0\\ 0 & 0 & 0 & 1 \end{array}\right).\;$$

We observe that none of these matrix squares is equal to the identity:

$${\left(S_{x}^{★}\right)}^{2}\;\neq \;I\;,\;{\left(S_{y}^{★}\right)}^{2}\;\neq \;I\;,\;{\left(S_{z}^{★}\right)}^{2}\;\neq \;I\;.\;$$

We have the calculation rule (A⊗B)(C⊗D)=(AC)⊗(BD), and we can therefore also write

$${\left(S_{x}^{★}\right)}^{2}=\frac{1}{2}\;\left(I+\sigma_{x}⊗\;\sigma_{x}\right)\;,\;\;{\left(S_{y}^{★}\right)}^{2}\;=\;\frac{1}{2}\;\left(I+\sigma_{y}⊗\;\sigma_{y}\right)\;,\;\;{\left(S_{z}^{★}\right)}^{2}\;=\;\frac{1}{2}\;\left(I+\sigma_{z}⊗\;\sigma_{z}\right).\;$$

Finally, given the relation $\;S_{2}={\left(S_{x}\right)}^{2}+{\left(S_{y}\right)}^{2}+{\left(S_{z}\right)}^{2}$, we have:

$$S_{2}^{★}\;=\;\left(\begin{array}{cccc} 2 & 0 & 0 & 0\\ 0 & 1 & 1 & 0\\ 0 & 1 & 1 & 0\\ 0 & 0 & 0 & 2 \end{array}\right)\;=\;\frac{1}{2}\;\left(3\;\;I+\sigma_{x}⊗\;\sigma_{x}+\sigma_{y}⊗\;\sigma_{y}+\sigma_{z}⊗\;\sigma_{z}\right).\;$$

If we introduce the “swap” operator ϖ *via* the relation

$$\varpi \;=\;\left(\begin{array}{cccc} 1 & 0 & 0 & 0\\ 0 & 0 & 1 & 0\\ 0 & 1 & 0 & 0\\ 0 & 0 & 0 & 1 \end{array}\right)\;,\;$$

we have

$$S_{2}^{★}\;=\;\varpi \;+\;I\;.\;$$

The swap operator ϖ is precisely the spin exchange operator introduced by Dirac in 1929 [28], which is fundamental, for example, to the explanation of magnetic phenomena arising from interacting spins.

### 4.4. Transforming a Product into a Sum

We seek to decompose the product space $\;\;\Sigma_{1/2}\;⊗\;\Sigma_{1/2}\;\;$ as a sum of spin spaces of the form $\;\Sigma_{j}$. To do this, we need to simultaneously diagonalize the operators $\;\;S_{z}^{★}\;\;$ and $\;\;S_{2}^{★}\;\;$ evaluated in the $\;\varphi_{i}\;$ basis introduced above by the relations that make explicit the matrices $\;S_{z}^{★}=\mathrm{diag}\;\left(1,\;0,\;0,\;-1\right)\;$ and $\;S_{2}^{★}=\mathrm{diag}\left(2,\;\left(\begin{array}{cc} 1 & 1\\ 1 & 1 \end{array}\right),\;2\right)$. This calculation is easy and is not detailed here. We then pose

$$\alpha \;=\;\frac{1}{\sqrt{2}}\;\;\left(e_{1}⊗\varepsilon_{2}\;\;-\;\;e_{2}⊗\varepsilon_{1}\;\right)\;\;$$

$$\beta_{+}\;=\;e_{1}⊗\varepsilon_{1}\;,\;\;\beta_{0}\;=\;\frac{1}{\sqrt{2}}\;\left(e_{1}⊗\varepsilon_{2}\;+\;e_{2}⊗\varepsilon_{1}\;\right)\;,\;\;\beta_{-}\;=\;e_{2}⊗\varepsilon_{2}\;.\;$$

We check that this is indeed the base of common eigenvectors that we are looking for: on the one hand, we have

$$S_{z}^{★}\;•\;\alpha \;=\;0\;,\;\;\;S_{2}^{★}\;•\;\alpha \;=\;0\;$$

and on the other hand

$$\left\{\begin{array}{c} S_{z}^{★}\;•\;\beta_{+}\;=\;\beta_{+}\;\;,\;S_{z}^{★}\;•\;\beta_{0}\;=\;0\;\;,\;S_{z}^{★}\;•\;\beta_{-}\;=\;-\beta_{-}\;\;,\;\\ S_{2}^{★}\;•\;\beta_{k}\;=\;2\;\beta_{k}\;\;,\;k\;\in \;\mu_{1}\;=\;\left\{+,\;0,\;-\right\}\;. \end{array}\right.\;$$

We call $\;\;\Sigma_{0}^{★}\;\;$ the space generated by the vector α, and we call $\;\;\Sigma_{1}^{★}\;\;$ the space generated by the family of $\;\;\beta_{k}\;,\;\;k\;\in \;\mu_{1}\;$ specified above. Then, the relations $\;S_{z}^{★}\;•\;\alpha =0$, $\;S_{2}^{★}\;•\;\alpha =0\;$ correspond to $\;S_{z}\;•\;e_{m}=0\;$ and $\;S_{2}\;•\;e_{m}=0$, i.e., zero spin. The relations $\;S_{z}^{★}\;•\;\beta_{k}\;=\;k\;\beta_{k}$, $\;S_{2}^{★}\;•\;\beta_{k}\;=\;2\;\beta_{k}\;$ (for k=−,0,+) are identical to $\;S_{z}\;•\;e_{m}\;=\;m\;e_{m}\;$ and $\;S_{2}\;•\;e_{m}\;=\;2\;e_{m}\;$ (m=−,0,+) for a unit spin. We have j=0 in the case of $\;\;\Sigma_{0}^{★}\;\;$, and we have j=1 for $\;\;\Sigma_{1}^{★}$. We therefore have the decomposition

$$\Sigma_{1/2}\;\;⊗\;\;\Sigma_{1/2}\;=\;\Sigma_{0}^{★}\;\;⊕\;\;\Sigma_{1}^{★}\;.\;$$

The interaction of two one-half spins provides a zero-spin space in direct sum with a unit-spin space.

### 4.5. Pauli Exclusion

We note that the vectors $\;\;\beta_{k}\;\;$ are symmetric combinations of the products $\;\;e_{j}⊗e_{k}\;\;$ in the case of two identical particles, where $\;\;\varepsilon_{k}\equiv e_{k}$. Pauli’s principle excludes these vectors as representing fermions if we do not take into account the spatial component of the wave function. In this particular case, only zero-spin $\;\;\Sigma_{0}^{★}\;\;$ space remains, and we can formally write the equation between spin spaces: $\;\;\frac{1}{2}\;⊕\;\frac{1}{2}\;\;=\;\;0$. It is important to note that the resulting state spanned by $\;\;\Sigma_{0}^{★}\;\;$ is the completely entangled singlet state α defined above.

## 5. Exchange and Indistinguishability

In the fractaquantum framework, the core principles of quantum mechanics—specifically, indistinguishability and the exchange of mediators—can describe the structure of social and artistic relations at a macroscopic scale.

The preceding reflections now push us to move forward in this enterprise; to seek out the sometimes surprising consequences of the fractaquantum hypothesis. We propose that indistinguishability can emerge in those phases of life where particular exchange behavior appears; such as, for example, in crowds. The manipulation of crowd behavior by totalitarian regimes prompts us to reflect on the nature of this possible interchangeability, which seems active on such occasions. Theoretical references on the subject go back to Le Bon [29].

Le Bon’s crowd is a new being in which every human component is reduced to a very primitive, animal-like component, to the benefit of strong links between the basic elements. Thus, “in a crowd every sentiment and act is contagious” and “the conscious personality has entirely vanished”. Who among us has not witnessed the power of “being together” in a stadium or demonstration? Le Bon’s work served as a starting point for Freud’s work on the same subject [30]. We believe there is a coherent link between crowd behavior and indistinguishability.

In standard quantum physics, elementary particles of the same nature are fundamentally indistinguishable. This is a legitimate “first-order” hypothesis for macroscopic entities like human beings. On a biological basis at a genetic level, two human beings are identical in their DNA sequence by more than 99%. Furthermore, during embryonic development, “totipotent” stem cells represent a phase of life characterized by total indistinguishability.

Social Atoms (with a capital A) are defined as inseparable elements of nature at any scale: in social contexts, when the specific identity of individuals becomes secondary to their collective behavior, they act as indistinguishable quantum-like entities.

While the “world is quantum”, even at our scale, our perception of “otherness” and uniqueness is viewed as a partial break in scale invariance, which creates individuation and potentially consciousness.

## 6. Quantum-like Applications

### 6.1. Social Sciences

An interesting social application of indistinguishability in fractaquantum, as mentioned before, is the analysis of the crowd. Drawing from Le Bon [29] and Freud [30], the crowd is described as a “new being” where the conscious personality vanishes. In a crowd, the “identity and interchangeability” of beings manifest as a macroscopic quantum property. Individuals become “contagious” in their sentiments and acts, effectively behaving like a coherent wave or a social laser [6,7]. Highly organized social structures like the army or the Church are analyzed as “artificial crowds” held together by strong internal links that suppress individual distinguishability.

If matter is defined by its constituents (fermions), structures are defined by the mediators (bosons) that link them. A structure is a composite set of Atoms linked together by “permanent chatter” through exchange bosons.

We have considered in Section 2 some examples of macroscopic mediators:

Conversation: A fundamental exchange that links two human “Atoms” into a temporary dyadic structure.

Dance and Love: These are viewed as relational mediators that create higher-order “loops” and permanence in social structures.

Libidinal Ties: Freud identifies the “libido” as the structuring element of crowds, acting as the exchange force that creates “group mind”.

Artworks like the “Duck–Rabbit” illustrate quantum cognition [31], providing information (measurement) forces the observer to choose one perception, effectively “reducing the wave packet” of the artistic meaning.

Measurement and Writing: The act of writing or filling out a questionnaire is interpreted as a “measurement” requested by a large Atom (society) from a small Atom (individual), forcing them to move from a state of anonymous indistinguishability to a finite, distinguishable set of components.

### 6.2. Performing Arts

The question of having a notation system for dance has been raised for at least several centuries. We refer here to the pioneering books of Arena [32] and Arbeau [33] in the 16th century. The famous Dancing Master of Playford [34] was re-edited 18 times between 1651 and 1728. In the same period, Feuillet, in his *Chorégraphie* [35], described the Beauchamp–Feuillet notation for ballroom and theatrical dances. In the 20th century, the notation system proposed by Laban [36] allows complex body movements to be codified using an algebraic approach.

Recently, Tripodi proposed in [37] to write the Tango. We can read: “The instructions of the person guiding and those of the person being guided are represented in the same line of writing, and sometimes in the same sign”, “In this sense, the writing takes into account the perspective and relationship between two people when they dance”, “The structural notation system of tango focuses fundamentally on the specificities of the movement of two bodies together”.

We have proposed the quantum modeling of a dancing couple in [38]. We have evocated the more intimate relationship between the body and the gaze, with dance, which evokes exchange, desire, and celebration: supported by music and a social setting, it allows for intimacy and physical contact; in doing so, it creates, by limiting itself here to so-called “couple” dances, an “intermediate being”, a stable and finite structure in time, which are admired in Waltz, Tango, Sevillana, or Rock and Roll competitions.

In a very convincing statement during the Ernst Strüngmann Forum on “Simplicity behind Absurdity: The Power of Quantum Thinking”, Svozil conveyed his idea that “Tango dancers are entangled” [8].

## 7. Conclusions

In this work, we suggest that quantum relations constitute a general theoretical framework in which meaning, logic, and social coherence emerge from operatorial, contextual, and fractal relational structures. Drawing inspiration from the fractaquantum hypothesis, eigenlogic, quantum semiotics, quantum-like social modeling, and relational interpretations of entanglement and individuality, we argue that meaning, logic, and social coherence emerge from relational eigenstructures rather than from classical object-based ontologies. The study of quantum relations shifts our focus from things to connections. By accepting that the world is quantum at all scales, we can begin to model human cognition, language, and social structures with the same precision and depth previously reserved for the atom.

Quantum relations offer a unifying conceptual foundation for understanding non-classical semantics across disciplines. Future work should investigate formal categorial structures and empirical applications of this relational paradigm.

## Data Availability

Data are contained within the article.
